# TMT-based proteomic profiling of serum reveals the impact of developmental stage and generation in beef cattle

**DOI:** 10.3389/fvets.2026.1723813

**Published:** 2026-03-09

**Authors:** Cunming Yang, Zhen Ma, Xiao Wang, Ullah Yaseen, Kexin Xu, Mingrui Fan, Xiangmin Yan, Zongsheng Zhao, Lei Chen

**Affiliations:** 1 College of Animal Science and Technology, Shihezi University, Shihezi, China; 2 Institute of Animal Husbandry, Xinjiang Academy of Animal Husbandry, Urumqi, China

**Keywords:** age effects, APOA4, HIST2H2AC, multigenerational selective breeding, TMT-based serum proteomics, Xinjiang Brown cattle (beef type)

## Abstract

**Introduction:**

Xinjiang Brown cattle are an important beef breed in Northwest China. Although multigenerational selective breeding has improved their growth performance, the accompanying molecular adaptations and potential physiological trade- ofs remain insufficiently elucidated at the systemic level. This study aimed to decipher the dynamic serum proteomic profiles shaped by both ontogeny and generational selection in Xinjiang Brown cattle, and to identify the associated key proteins and pathways.

**Methods:**

Serum samples from 18 bulls across three genera- tions (A, B, C) at 3 and 9 months of age were analyzed using Tandem Mass Tag (TMT)-based quantitative proteomics.

**Results:**

Under stringent quality control (FDR < 1%), 583 high-confidence proteins were identified. Diferentially expressed proteins (DEPs) were screened using thresholds of |fold change| ≥ 1.2 and *p*-value < 0.05. Bioinformatic analysis was performed to elucidate protein functions, and 12 key DEPs were validated by Parallel Reaction Monitoring (PRM). Serum pro- teome variations were primarily driven along two dimensions: age and genera- tion. Age-related diferences, marked by upregulated proteins such as APOA4 at 3 months, were enriched in lipid metabolism and skeletal development pathways. In contrast, generational shifts revealed a coordinated downregulation of proteins involved in immune-vascular homeostasis, including VWF, THBS1, TGFB1, CCN2, and TIMP3, alongside an increase in the chromatin regulator HIST2H2AC. Enrichment analysis highlighted “platelet activation” and the “TGF-beta signal- ing pathway” as significantly altered intergenerational regulatory networks. PRM validation confirmed the reliability of the proteomic data.

**Discussion:**

This study reveals that the breeding strategy for Xinjiang Brown cattle prioritizes shaping a proteomic landscape that promotes growth and metabolism, potentially at the cost of atten- uated immune-vascular reactivity. The identified panel of candidate proteins pro- vides a molecular framework for evaluating breeding outcomes and designing balanced selection strategies. Follow-up research should further investigate the functions of these candidate proteins and validate their predictive value for health and production performance in independent herds.

## 1 Introduction

The beef cattle industry holds strategic importance in securing animal protein supply and promoting sustainable agricultural development (1). China, as the world’s third-largest beef producer, achieved an output of 8.04 million tons in 2022, representing 9.7% of global production (2). Xinjiang, a key beef-producing region in northwestern China, is home to the indigenous Xinjiang Brown cattle. This breed is well-adapted to the local environment, exhibiting notable stress resilience and robust performance. This breed now constitutes approximately 20% of Xinjiang’s total cattle population (3–5). Through multiple generations of selective breeding, Xinjiang Brown cattle have been improved for growth, meat quality, and environmental adaptability. However, accelerated genetic improvement may pose biological challenges. One of the major concerns in genetic improvement programs is the accumulation of harmful recessive mutations, which may lead to serious genetic disorders. For instance, The CD18 D128G mutation is known to cause leukocyte adhesion deficiency (LAD) in Holstein bulls (6). Similarly, a 3.3 kb deletion in the FANCI gene leads to brachyspina syndrome, which occurs rarely in live births (1 in 105), but causes significant embryonic loss (7). Some mutations, like the highly conserved SUGT1 p. W317R, can even be embryonic lethal yet often go undetected during routine screening (8). These examples highlight the need for deeper genetic surveillance and monitoring during breeding programs. Concurrently, metabolic variations across developmental stages necessitate precise nutritional management. Serum proteomic studies indicate that around 9 months of age, cattle undergo a metabolic shift from catabolism to anabolism (9). This transition alters nutrient partitioning and creates opportunities for targeted dietary interventions. Importantly, the systemic physiological effects of nutritional interventions, including the modulation of antioxidant and immune pathways, are detectable in the blood proteome—a principle that is conserved across livestock species. For instance, dietary supplementation with bioactive compounds like *Litsea cubeba* essential oil in pigs has been shown to enhance serum antioxidant markers (e.g., catalase) and nutrient digestibility, directly linking feed additives to circulating biomarkers and improved performance (10). Similarly, studies in broilers demonstrate that specific tannins can simultaneously enhance growth, serum antioxidant capacity (e.g., T-AOC, GSH-Px), and immune parameters (e.g., immunoglobulins, anti-inflammatory cytokines), illustrating a consistent nexus between optimized nutrition, immune modulation, and systemic physiological remodeling (11). This underscores that functional feed additives can systemically influence key physiological states, a concept further supported by research linking dietary factors to measurable shifts in systemic antioxidant status and development (12). Consistent with this principle, early supplementation with rumen-bypass lipids (RBL) has been shown to significantly improve fat deposition in the longissimus dorsi muscle (13). These stage-dependent metabolic dynamics underscore the importance of precision nutrition in modern cattle farming. Therefore, investigating how selective breeding influences the genetic load in Xinjiang Brown cattle, and how this genetic background interacts with both metabolic shifts and responsive nutritional strategies, holds significant scientific value. Such research can inform the development of superior breeding schemes, optimize feeding regimens, and help mitigate the propagation of deleterious genetic traits.

Proteomics offers a powerful way to explore these questions. It allows researchers to examine how proteins are expressed, modified, and connected in biological networks, offering insights into development, metabolism, and disease mechanisms (14, 15). Serum proteomics is particularly valuable as it relies on non-invasive blood sampling and enables near real-time monitoring of physiological status. Using high-resolution mass spectrometry, it becomes possible to identify and quantify changes in serum proteins with great precision, helping track dynamic biological changes over time. While this approach has been widely applied in human medicine to identify disease biomarkers and elucidate pathological mechanisms, its value in livestock science is increasingly recognized (16–18). For instance, in animal studies, Deng et al. (19) discovered important regulators of early embryo development, such as GRN and FGL1, in the serum of pregnant donkeys, demonstrating the utility of serum proteomics for monitoring reproductive physiology. More directly relevant to this study, serum proteomic analyses in cattle have been successfully used to investigate metabolic adaptations, health status, and traits related to productivity, thereby establishing a solid methodological precedent for our investigation of generational protein expression dynamics in Xinjiang Brown cattle.

Based on this foundation, we collected blood samples from Xinjiang Brown bulls (beef type) across three generations and two key developmental stages (3 and 9 months of age). Employing Tandem Mass Tag (TMT) proteomics, we identified serum proteins exhibiting variations dependent on age and generational lineage, followed by comprehensive bioinformatic functional analysis. This study aims to reveal dynamic changes in the serum proteome associated with genetic selection and age-related physiological shifts, elucidate the impacts of genetic background and developmental stage on immune function, metabolic regulation, and growth trajectories, and thereby provide a scientific foundation for optimizing breeding strategies and enhancing production efficiency.

## 2 Materials and methods

### 2.1 Sample collection and grouping

All animal procedures were carried out in accordance with ethical standards and approved by the Institute of Animal Science, Xinjiang Academy of Animal Sciences (Xinjiang, China, Approval No. JXM-KX-2024001). This study used healthy beef-type Xinjiang Brown bulls provided by the Xinjiang Yili Yixin Livestock Farming Cooperative. Bulls were selected from three different generations (A), (B), and (C). For each generation, we included three bulls aged 3 months and three bulls aged 9 months, resulting in a total of 18 animals. Based on generation and age, the animals were divided into six experimental groups: A3, A9, B3, B9, C3, and C9. Blood samples (5 mL each) were collected from all animals before morning feeding to avoid diet-related variation. Blood samples were collected without anticoagulants and allowed to clot naturally. Subsequently, they were centrifuged at 3,500 rpm for 10 min. The resulting serum supernatant was then carefully separated and stored at −80 °C for subsequent analysis.

### 2.2 Protein extraction and digestion

Protein extraction was carried out using Bio-Rad ProteoMiner beads (Catalog No. 1633007). The beads were transferred into new 1.5 mL centrifuge tubes, and 200 μL of 1 × PBS was added. After pipetting to mix, the tubes were centrifuged at 6,000 × g for 1 min, and the supernatant was discarded. This washing step was repeated three times to ensure bead purification. Next, 100 μL of serum was added to the cleaned beads, and the mixture was inverted using a vertical mixer for 2 h at room temperature. After incubation, the tubes were centrifuged, and the supernatant was discarded. Then, 500 μL of 1 × PBS was added, shaken for 5 min, and centrifuged again at 6,000 × g for 1 min. This wash step was also repeated three times. For protein elution, 100 μL of 1% trifluoroacetic acid (TFA) was added. The mixture was shaken for 10 min, centrifuged at 6,000 × g for 1 min, and the supernatant was transferred to a new 1.5 mL centrifuge tube. This elution was repeated once, and the two eluates were combined. The combined eluate was then lyophilized (freeze-dried) and resuspended in 50 μL of 8 M urea buffer (UA). A 10 μL aliquot was taken for protein quantification using a colorimetric assay with a serial dilution of bovine serum albumin as the standard (see Supplementary Figure 1A for the standard curve, *R*^2^ = 0.993). The quality and integrity of the extracted proteins were verified by SDS-PAGE (Supplementary Figure 1B). Based on the measured concentration, an aliquot containing exactly 100 μg of total protein was accurately dispensed from each sample for digestion. This input amount was selected to ensure abundant peptide yield for the subsequent TMTpro 18plex labeling and LC–MS/MS analysis, which is a standard and robust starting quantity in quantitative proteomics to achieve high-depth coverage and reliable quantification. Proteins were reduced by adding 5 mM dithiothreitol (DTT) and incubating at 37 °C for 1 h. Next, samples were alkylated with 10 mM iodoacetamide (IAA) at room temperature in the dark for 30 min. The sample was diluted 4 times by adding 50 mM ammonium bicarbonate (ABC) buffer, followed by digestion with trypsin at a 1:50 enzyme-to-protein ratio. The digestion mixture was incubated overnight at 37 °C. Finally, all samples were desalted using C18 cartridges to remove residual urea. The desalted peptides were dried using vacuum centrifugation and stored for further analysis.

### 2.3 TMT labeling and peptide fractionation

Prior to TMT labeling, all peptide samples were fully randomized to minimize potential batch effects and technical variability. Each peptide sample was labeled using TMTpro 18plex reagents (Thermo Fisher Scientific, Cat. No. A52045), following the manufacturer’s instructions. After labeling, the peptides were fractionated using basic reversed-phase chromatography on a RIGOL L-3000 dual-gradient high-performance liquid chromatography (HPLC) system, equipped with an Agela Durashell C18 column (4.6 × 250 mm i.d., 5 μm, 100 A). the column oven was maintained at 45 °C.

Before sample loading, the C18 column was pre-washed with 100% methanol, then equilibrated with Buffer A (0.1% ammonium hydroxide and 2% acetonitrile). Peptide mixtures were injected into the column and separated using a gradient from mobile phase A (2% acetonitrile, 0.1% ammonium hydroxide) to mobile phase B (98% acetonitrile, 0.1% ammonium hydroxide) over a 72 min run at a flow rate of 0.7 mL/min. The solvent gradient was set as follows: 5%B, 0 min; 5–8% B, 5 min; 8–18% B, 30 min; 18–32% B, 27 min; 32–95% B, 6 min; 95–5%, 4 min. The collected fractions were orthogonally concatenated into 10 pooled fractions for further LC–MS/MS analysis.

### 2.4 Peptide identification by nano UPLC–MS/MS

Nanoflow LC–MS/MS analysis of tryptic peptides was performed using a Q Exactive HF-X quadrupole-Orbitrap mass spectrometer (Thermo Fisher Scientific, Bremen, Germany) coupled online to an EASY-nLC 1,200 UPLC system via a nano-electrospray ion source. Approximately 500 ng of peptides were loaded onto a 25 cm-long analytical column (150 μm inner diameter), packed with ReproSil-Pur C18-AQ, 1.9 μm silica beads (Beijing Qinglian Biotech Co., Ltd., Beijing, China). Column temperature was maintained at 60 °C using a custom-built column oven. Peptide separation was carried out using a solvent gradient at a flow rate of 600 nL/min. Mobile phase A: 0.1% formic acid in water, Mobile phase B: 80% acetonitrile (ACN) with 0.1% formic acid in water. The gradient conditions were as follows:7 to 15% B over 7 min, 15 to 25% B over 27 min, 25 to 40% B over 15 min, Followed by a final wash at 95% B for 11 min. The mass spectrometer was operated in data-dependent acquisition (DDA) mode using a “top-40” method. This setting was selected to achieve deep proteome coverage from the complex serum samples, leveraging the instrument’s rapid scanning speed to maximize identifications across a wide dynamic range while maintaining quantitative precision within the chromatographic peak width. Full MS scans were acquired in the Orbitrap 60,000 resolution (m/z 407–1,500) with an automatic gain control (AGC) target of 3e^6^ and a maximum injection time of 20 ms. The most intense ions were isolated with a 1.0 m/z window and fragmented via higher-energy collisional dissociation (HCD) at 34% normalized collision energy (NCE). MS/MS spectra were acquired at 45,000 resolution with an AGC target of 5e^4^ and a maximum injection time of 86 ms. A dynamic exclusion of 16 s was applied to prevent repeated sequencing.

### 2.5 Parallel reaction monitoring

To independently validate the differential expression trends identified in the TMT-based discovery proteomics, parallel reaction monitoring (PRM) analysis was conducted. Target peptides were selected from the pool of differentially expressed proteins (DEPs) generated by the TMT analysis. A total of twelve DEPs were chosen for targeted verification, comprising five proteins that showed significant variation across age groups and seven proteins that varied across generational groups, ensuring coverage of key comparative contexts from the discovery phase.

To validate the TMT-based quantification results, parallel reaction monitoring (PRM) analysis was conducted. Peptide samples were first dissolved in 100 μL of mobile phase A, then centrifuged at 14,000 × g for 20 min. The resulting supernatant was subjected to LC separation using the following gradient: 0–5 min: 5–8% B; 5–35 min: 8–18% B; 35–62 min: 18–32% B; 62–64 min: 32–95% B; 64–68 min: 95% B; 68–72 min: 5% B. The separation was performed at a flow rate of 0.7 mL/min. Eluted fractions were pooled into three combined fractions, freeze-dried, and stored at −80 °C for later analysis.

Mobile phases were prepared as follows: Mobile phase A: 100% water with 0.1% formic acid; Mobile phase B: 80% acetonitrile with 0.1% formic acid. Freeze-dried peptides were resuspended in 10 μL of mobile phase A, centrifuged at 14,000 × g for 20 min at 4 °C, and 1 μg of the supernatant was injected for LC–MS/MS analysis. LC–MS/MS analysis for PRM validation was carried out using a Q Exactive HF-X mass spectrometer (Thermo Fisher Scientific) coupled with a Nanospray Flex™ (NSI) ion source. The gradient for chromatographic separation was set as follows: 0–5 min: 8–12% B; 5–35 min: 12–30% B; 35–44 min: 30–40% B; 44–45 min: 40–95% B; 45–60 min: 95% B. MS parameters (Q Exactive HF-X): Ion source: Nanospray Flex™ (NSI); Spray voltage: 2.2 kV; Capillary temperature: 320 °C; Full MS scan range: 350–1,500 m/z; MS1 resolution: 120,000 (@ 200 m/z); MS2 resolution: 15,000 (@ 200 m/z); HCD collision energy: 27%; MS1 AGC target: 3e6; Max. injection time: 80 ms; MS2 AGC target: 5e4; Max. injection time: 45 ms. Raw data files (.raw) were processed using Skyline software (v.21.1+) for quantification and validation.

### 2.6 Statistical analysis

Differentially expressed proteins (DEPs) were identified using thresholds of fold change (FC) ≥ 1.2 or ≤ 0.83 combined with a *p*-value < 0.05 for statistical significance. Database searching was performed using Proteome Discoverer software (version 2.4, Thermo Fisher Scientific, United States) with the following parameters: precursor mass tolerance of 10 ppm, fragment mass tolerance of 0.02 Da, and a maximum of two missed cleavages. The false discovery rate (FDR) was set at 1%. The biological functions of DEPs were annotated using Gene Ontology (GO) and Kyoto Encyclopedia of Genes and Genomes (KEGG) pathway analyses, conducted via the R package (20). For pattern analysis, DEP clustering was performed using the “Complex Heatmap” package in R. To quantitatively evaluate the separation between predefined biological groups in the clustering results, the silhouette coefficient was computed using the cluster package in R. To explore functional relationships, protein–protein interaction (PPI) networks were constructed using the STRING database (v12.0), applying a high-confidence interaction score >0.7. Differential PPI networks were further generated in R using the igraph package and visualized with supporting libraries including ggraph, dplyr, and RColorBrewer.

## 3 Results

### 3.1 Overview of protein quality and sequencing results

**Figure 1:**
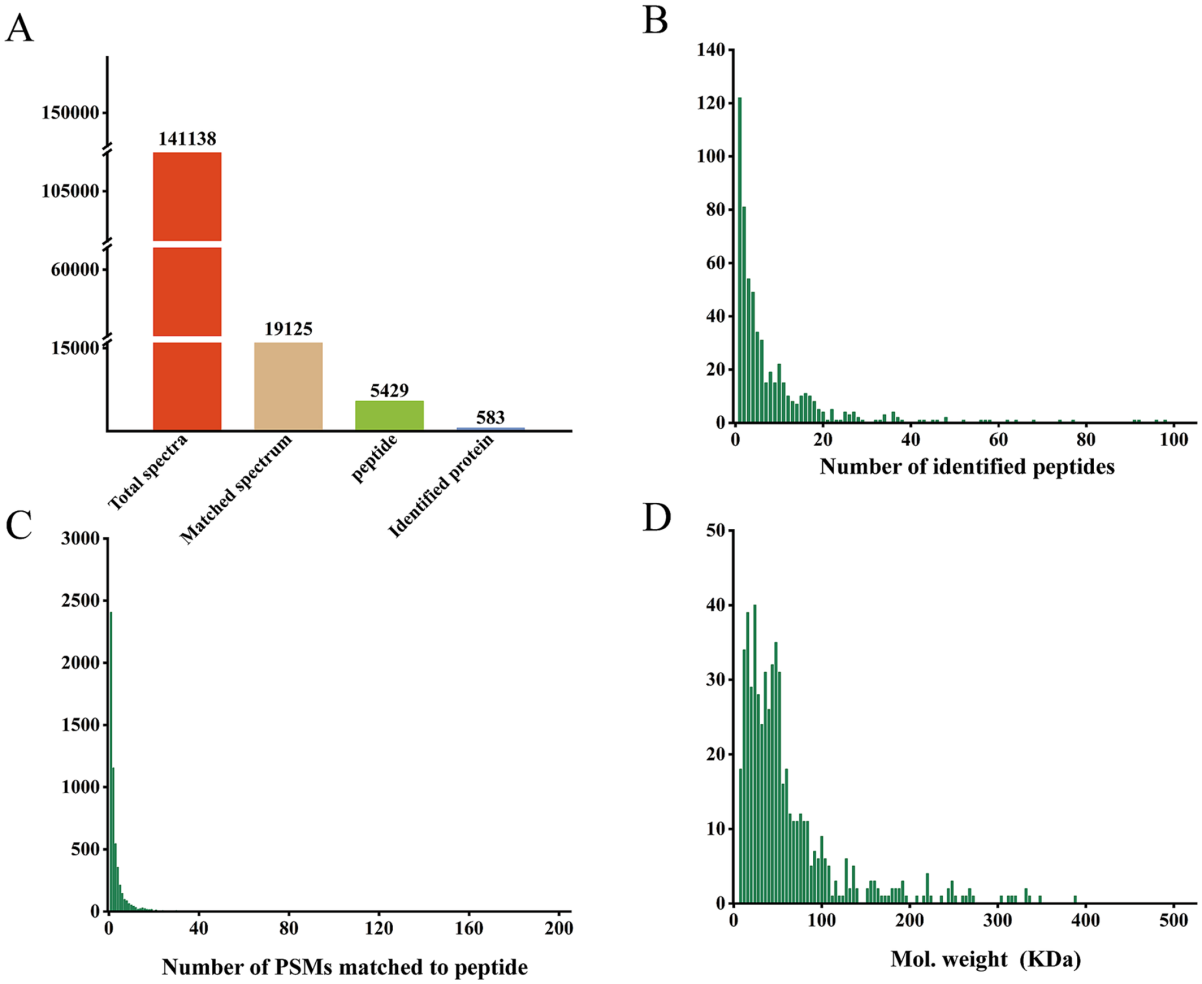
Basic proteomic profiling statistics. **(A)** Summary of protein identification results; **(B)** distribution of peptide counts per identified protein; **(C)** frequency distribution of peptide-spectrum matches (PSMs); **(D)** molecular weight distribution of detected proteins.

Protein concentrations in all samples were quantified (Supplementary Table S1). A standard curve was plotted, yielding an *R*^2^ value of 0.993, which indicating excellent linearity (Supplementary Figure 1A). SDS-PAGE analysis revealed clear protein bands without degradation (Supplementary Figure 1B), confirming sample integrity for downstream analysis. During LC–MS/MS analysis, a total of 141,138 secondary spectra were identified, of which 19,125 were valid spectra. From these, 5,429 peptides and 583 unique proteins were identified (Figure 1A). Further key distribution characteristics showed the following: Most proteins were identified with 1 to 15 peptides (Figure 1B), demonstrating high identification efficiency. Peptide-spectrum matches (PSMs) were primarily distributed in the range of 1–21 (Figure 1C). Most Proteins had molecular weights in the range of 0–100 kDa (Figure 1D). Overall, the mass spectrometry results met quality standards and were suitable for subsequent analyses.

### 3.2 Identification and differential expression analysis of proteins

**Figure 2:**
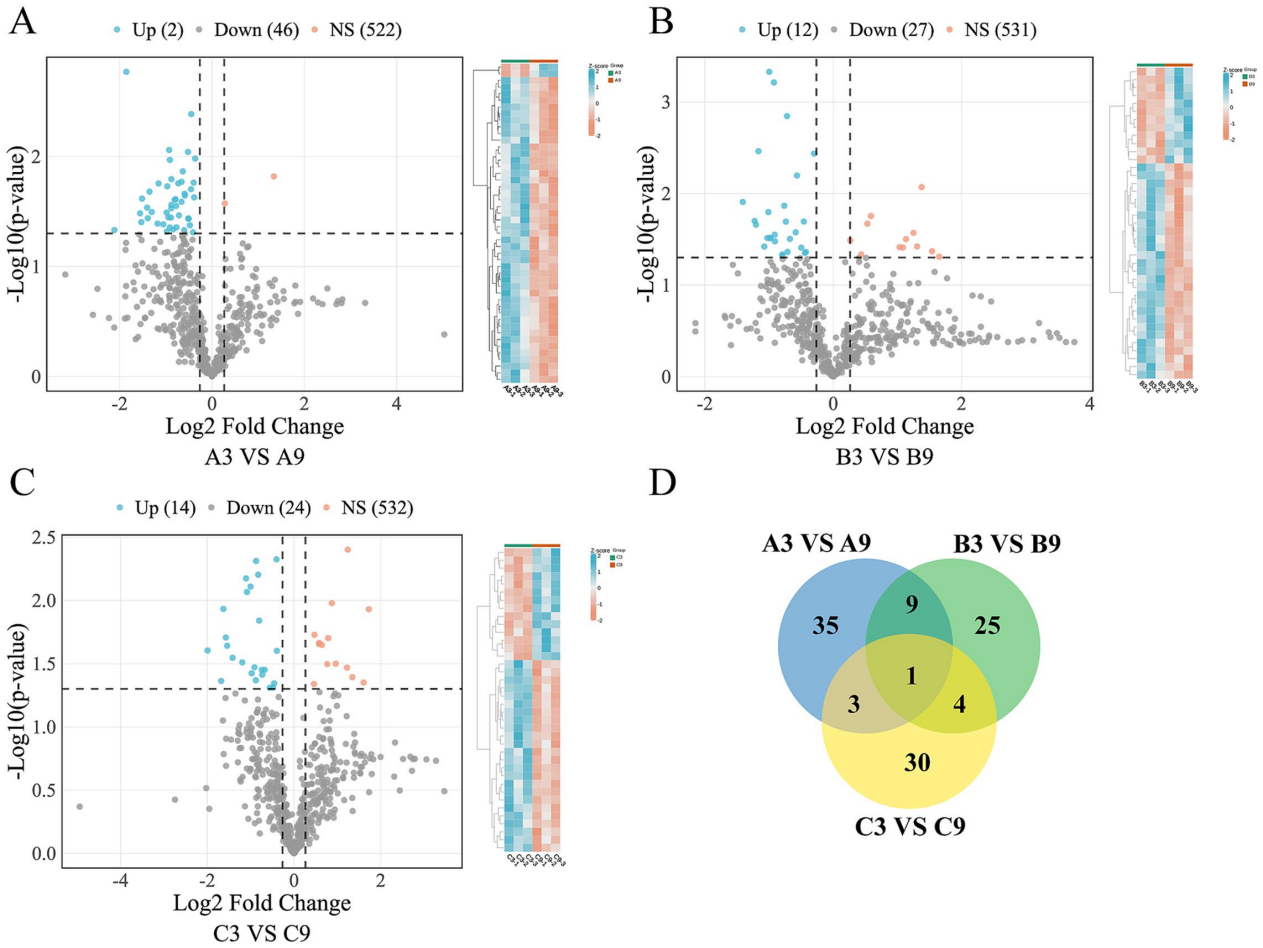
Differential protein expression patterns across age groups. Volcano plots **(A–C)** depict differentially expressed proteins (DEPs) between comparative age groups, with corresponding hierarchical clustering heatmaps. Red and blue data points denote up- and down-regulated proteins, respectively. Vertical dashed lines indicate the 1.2-fold change (FC) threshold, while horizontal dashed lines represent the significance level (*p* = 0.05; consistent throughout). Hierarchical relationships of age-specific DEPs are illustrated in **(D)**.

A Comparative analysis of differentially expressed proteins (DEPs) across age and generational groups in Xinjiang Brown cattle revealed that the 9-month-old first generation (A9 vs. A3) exhibited 48 DEPs (2 upregulated, 46 downregulated, Figure 2A), the second generation (B9 vs. B3) showed 39 DEPs (12 upregulated, 27 downregulated, Figure 2B), and the third generation (C9 vs. C3) had 38 DEPs (14 upregulated, 24 downregulated, Figure 2C) compared to the 3-month-old groups. The intersection analysis identified 10 shared DEPs between A9 vs. A3 and B9 vs. B3 comparisons, 4 shared DEPs between A9 vs. A3 and C9 vs. C3, and 5 shared DEPs between B9 vs. B3 and C9 vs. C3. Only one protein, APOA4 (UniProt ID: F1N3Q7) was common to all three age comparisons (Figure 2D).

**Figure 3:**
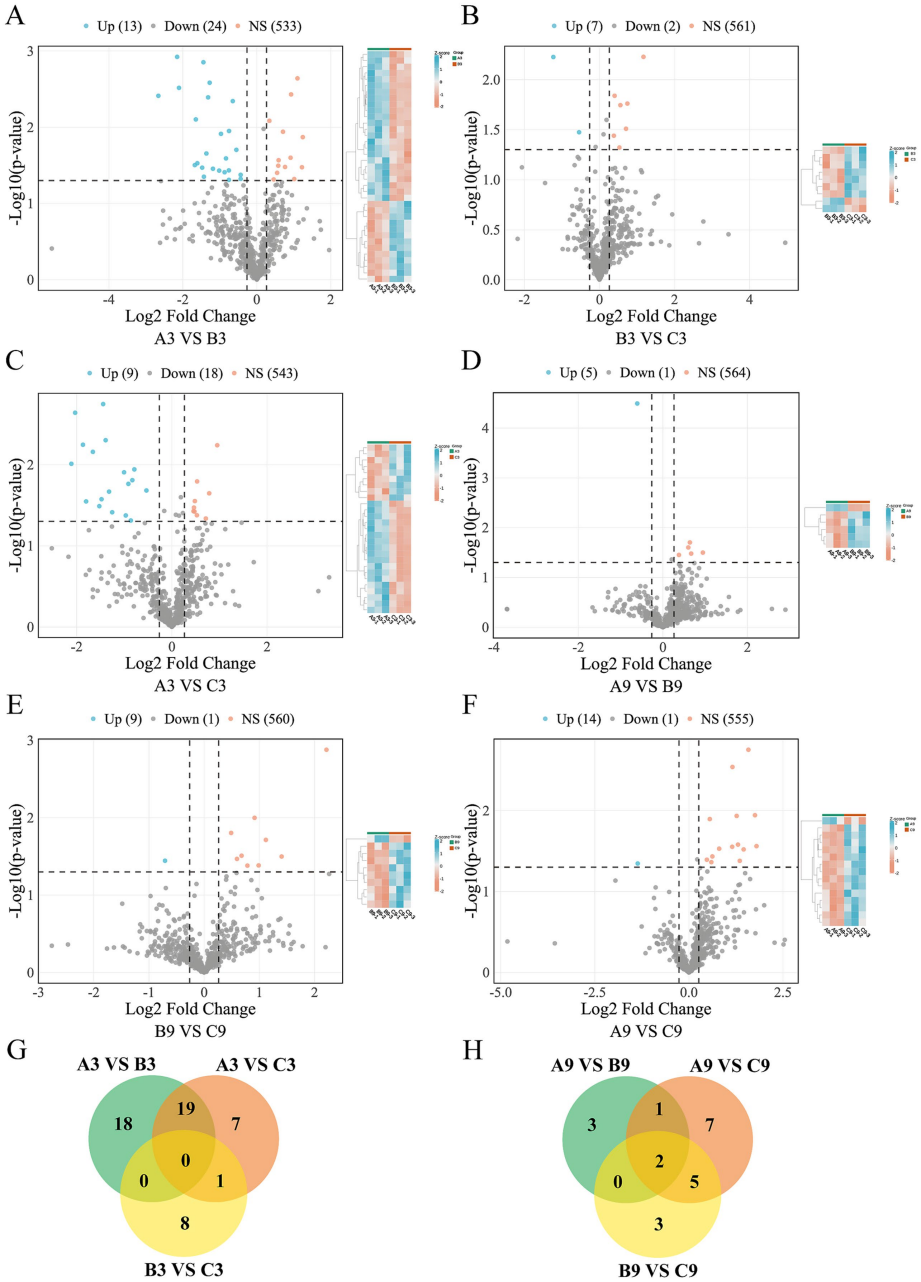
Differential protein expression patterns across generation. **(A–C)** Volcano plots and corresponding clustering heatmaps depict differentially expressed proteins (DEPs) between generational groups at 3 months of age. **(D–F)** Parallel analyses showing DEPs and clustering heatmaps at 9 months of age. **(G)** Hierarchical relationships of DEPs across generations in the 3-month cohort. **(H)** Hierarchical relationships of DEPs across generations in the 9-month cohort.

To investigate generational genetic effects, a comparative analysis across generations was conducted at 3 and 9 months. At 3 months, the comparisons revealed 37 DEPs (13 upregulated, 24 downregulated) in B3 vs. A3 (Figure 3A), 27 DEPs (9 upregulated, 18 downregulated) in C3 vs. A3 (Figure 3B), and 9 DEPs (7 upregulated, 2 downregulated) in C3 vs. B3 (Figure 3C). Venn analysis revealed 19 shared DEPs between B3 vs. A3 and C3 vs. A3, no overlap between B3 vs. A3 and C3 vs. B3, and 1 shared DEP between C3 vs. A3 and C3 vs. B3. There were no common DEPs in any of the three comparisons (Figure 3G).

At 9 months, the analysis identified 6 DEPs (5 upregulated, 1 downregulated) in B9 vs. A9 (Figure 3D), 15 DEPs (14 upregulated, 1 downregulated) in C9 vs. A9 (Figure 3E), and 10 DEPs (9 upregulated, 1 downregulated) in C9 vs. B9 (Figure 3F). The intersections included 3 shared DEPs between B9 vs. A9 and C9 vs. A9, 2 between B9 vs. A9 and C9 vs. B9, and 7 between C9 vs. A9 and C9 vs. B9, with two common DEPs conserved across all generational comparisons: A1A4R1 (HIST2H2AC/histone H2A type 2-C) and F1ML72 (RPL34/large ribosomal subunit protein eL34) (Figure 3H).

Hierarchical clustering analysis visualized the expression patterns of differentially expressed proteins (DEPs) across age and generational groups (Figures 2A–C, 3A–F). To objectively assess the intergroup separation observed in the heatmaps, the silhouette coefficient was calculated. Quantitative analysis confirmed good separation across all predefined biological groups, with the A9 vs. B9 comparison demonstrating particularly strong distinctiveness (Supplementary Table S2). Collectively, the consistently positive silhouette coefficients support high within-group consistency and distinct intergroup expression profiles, thereby validating the reliability of the DEPs identified in this study.

### 3.3 GO enrichment analysis

#### 3.3.1 GO enrichment analysis of age-related differences

**Figure 4:**
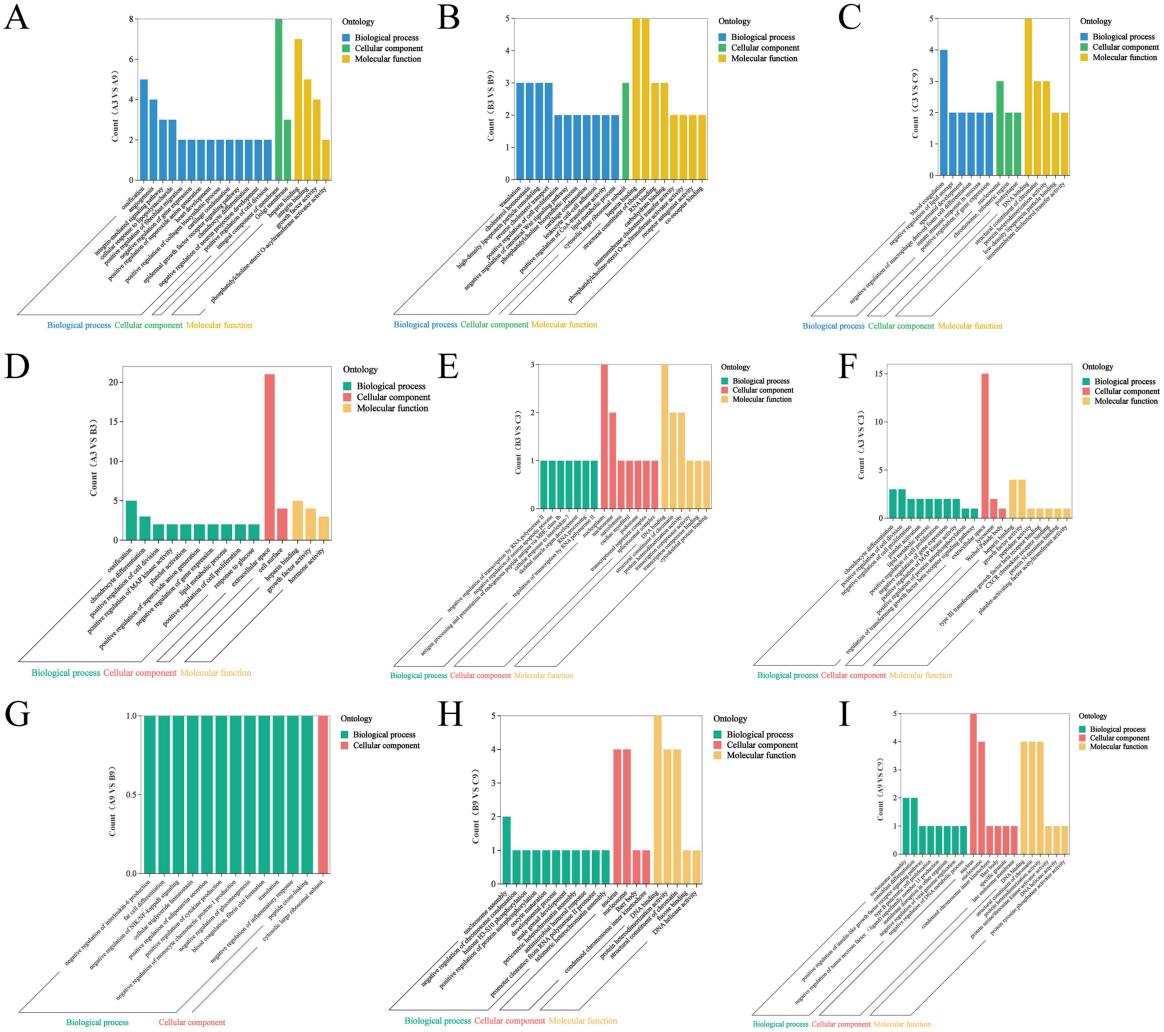
Gene ontology (GO) enrichment analysis of differentially expressed proteins (DEPs) in Xinjiang brown cattle across age groups and generations. **(A–I)** Represent GO enrichment results for DEPs identified in distinct comparisons: age-group comparisons (A3 vs. A9, B3 vs. B9, C3 vs. C9) and generational comparisons at 3 months (A3 vs. B3, A3 vs. C3, B3 vs. C3) and 9 months (A9 vs. B9, A9 vs. C9, B9 vs. C9). The X-axis denotes GO terms, while the Y-axis indicates the corresponding gene count.

GO enrichment analysis of differentially expressed proteins (DEPs) across age groups revealed distinct biological processes: In A9 vs. A3 comparisons (48 DEPs, 406 GO terms, 27 significant, BP,21, CC:2, MF:4), key enrichments included skeletal development (GO:0001503) and angiogenesis (GO:0001525) in biological processes; Golgi membrane (GO:0000139) in cellular components; and growth factor activity (GO:0008084) in molecular functions (Figure 4A). For B9 vs. B3 (39 DEPs, 268 terms, 25 significant, BP,16, CC,1, MF:8), significant terms comprised translation (GO:0006412), chondrocyte differentiation (GO:0002062), angiogenesis (GO:0001525), cholesterol metabolism (GO:0008203), and cell proliferation regulation (GO:0008284) in BP; cytoplasmic ribosomal subunit (GO:0022625) in CC; and ribosomal structural constituent (GO:0003735) with RNA binding (GO:0003723) in MF (Figure 4B). In C9 vs. C3 (38 DEPs, 273 terms, 14 significant, BP,6, CC,3, MF:5), enrichments featured negative lipid storage regulation (GO:0010888), negative sperm development regulation (GO:0007286), and mucosal innate immunity (GO:0002227) in BP; telomeric region (GO:0000781), nucleosome (GO:0000786), and postsynapse (GO:0098794) in CC; plus DNA binding (GO:0003677) and membrane cholesterol transfer (GO:0120020) in MF (Figure 4C).

#### 3.3.2 GO enrichment analysis of generational differences

To assess genetic background influences, GO analysis was performed on generational DEPs at 3 and 9 months.

At 3 months: GO analysis of generational comparisons revealed B3 vs. A3 (37 DEPs; 330 terms; 13 significant) enriched in skeletal development (GO:0001503), chondrocyte differentiation (GO:0002062), cell division regulation (GO:0051781), and lipid metabolism (GO:0006629) for biological processes (BP); extracellular space (GO:0005615) and cell surface (GO:0009986) for cellular components (CC); plus growth factor activity (GO:0008084) and hormone binding (GO:0005130) for molecular functions (MF) (Figure 4D). C3 vs. A3 (27 DEPs; 257 terms; 96 significant) featured cell proliferation (GO:0051781) and skeletal development (GO:0001503) in BP; extracellular space (GO:0005615) and lysosomes (GO:0005764) in CC; with growth factor activity (GO:0008084) and heparin binding (GO:0008017) in MF (Figure 4E). C3 vs. B3 (9 DEPs; 41 terms; 24 significant) showed chromatin/gene regulation (GO:0006396), developmental control (GO:0060538), and immune response (GO:0098761) in BP; nucleoplasm (GO:0005654) and nucleosomes (GO:0000786) in CC; alongside DNA binding (GO:0003677) and chromatin structure (GO:0030527) in MF (Figure 4F).

At 9 months: B9 vs. A9 (6 DEPs; 32 terms; 13 significant) demonstrated immune-metabolic regulation including suppressed IL-6 production (GO:0032715), anti-inflammation (GO:0050728), and adipocyte differentiation (GO:0045444) in BP, with ribosomal subunits (GO:0022625) in CC (Figure 4G). C9 vs. A9 (15 DEPs; 132 terms; 55 significant) highlighted skeletal regulation, viral response, and cellular homeostasis in BP; nucleosomes (GO:0000786), nuclear organization (GO:0005634), and centromere structure (GO:0000939) in CC; plus DNA binding (GO:0003677) in MF (Figure 4H). C9 vs. B9 (10 DEPs; 76 terms; 41 significant) involved chromatin remodeling, reproductive control, and immune processes in BP; shared CC enrichments (nucleosomes, nucleus, centromeres); with growth factor receptor binding (GO:0070851) added to core MF terms (Figure 4I).

Age-driven changes centered on skeletal and metabolic pathways, while generational shifts predominantly involved immune modulation, gene regulation, and proliferative control, underscoring genetic selection impacts.

### 3.4 KEGG pathway enrichment analysis

**Figure 5:**
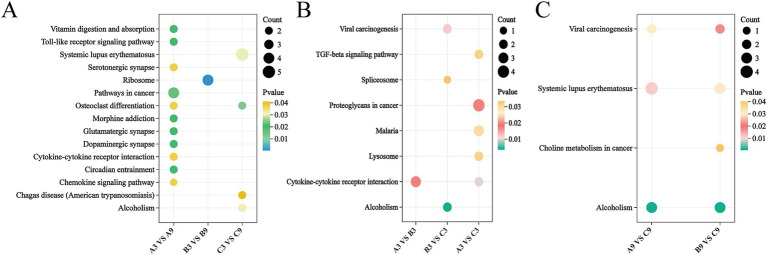
KEGG pathway analysis of differentially expressed proteins (DEPs) in Xinjiang brown cattle across age groups and generations. **(A–C)** Enrichment analysis of DEP-associated pathways: **(A)** Age-group comparisons (3-month vs. 9-month), **(B)** generational comparisons at 3 months, and **(C)** generational comparisons at 9 months. The X-axis represents comparison groups, while the Y-axis indicates pathway enrichment significance (−log₁₀[*p*-value]). Dot size corresponds to the number of DEPs per pathway, and dot color reflects the *p*-value of enrichment.

KEGG pathway analysis of DEPs across nine comparison groups revealed: Age-related comparisons (A3 vs. A9, B3 vs. B9, C3 vs. C9) exhibited 11, 1, and 4 significantly enriched pathways respectively, primarily involving vitamin digestion/absorption (ko04977), osteoclast differentiation (ko04380), ribosome (ko03010), and systemic lupus erythematosus (ko05322) (Figure 5A). Generational comparisons at 3 months (A3 vs. B3, B3 vs. C3, A3 vs. C3) showed 1, 3, and 5 enriched pathways, dominated by cytokine-cytokine receptor interaction (ko04060), TGF-beta signaling (ko04350), alcoholism (ko05034), and spliceosome (ko03040) (Figure 5B). Generational comparisons at 9 months (A9 vs. B9, B9 vs. C9, A9 vs. C9) had 0, 3, and 4 enriched pathways, featuring alcoholism (ko05034), viral carcinogenesis (ko05203), and systemic lupus erythematosus (ko05322) (Figure 5C).

This analysis indicates that age transitions correlate strongly with metabolic, immune, and skeletal development pathways. The immune regulation, cell signaling, and metabolic control pathways enriched in 3-month generational comparisons suggest their pivotal roles in immune system maturation and adaptive regulation. Despite fewer DEPs at 9 months, enriched pathways remained linked to immune/metabolic processes and pathologies, particularly systemic lupus erythematosus, implying significant intergenerational divergence in environmental stress response and disease susceptibility.

### 3.5 Validation using parallel reaction monitoring

**Figure 6:**
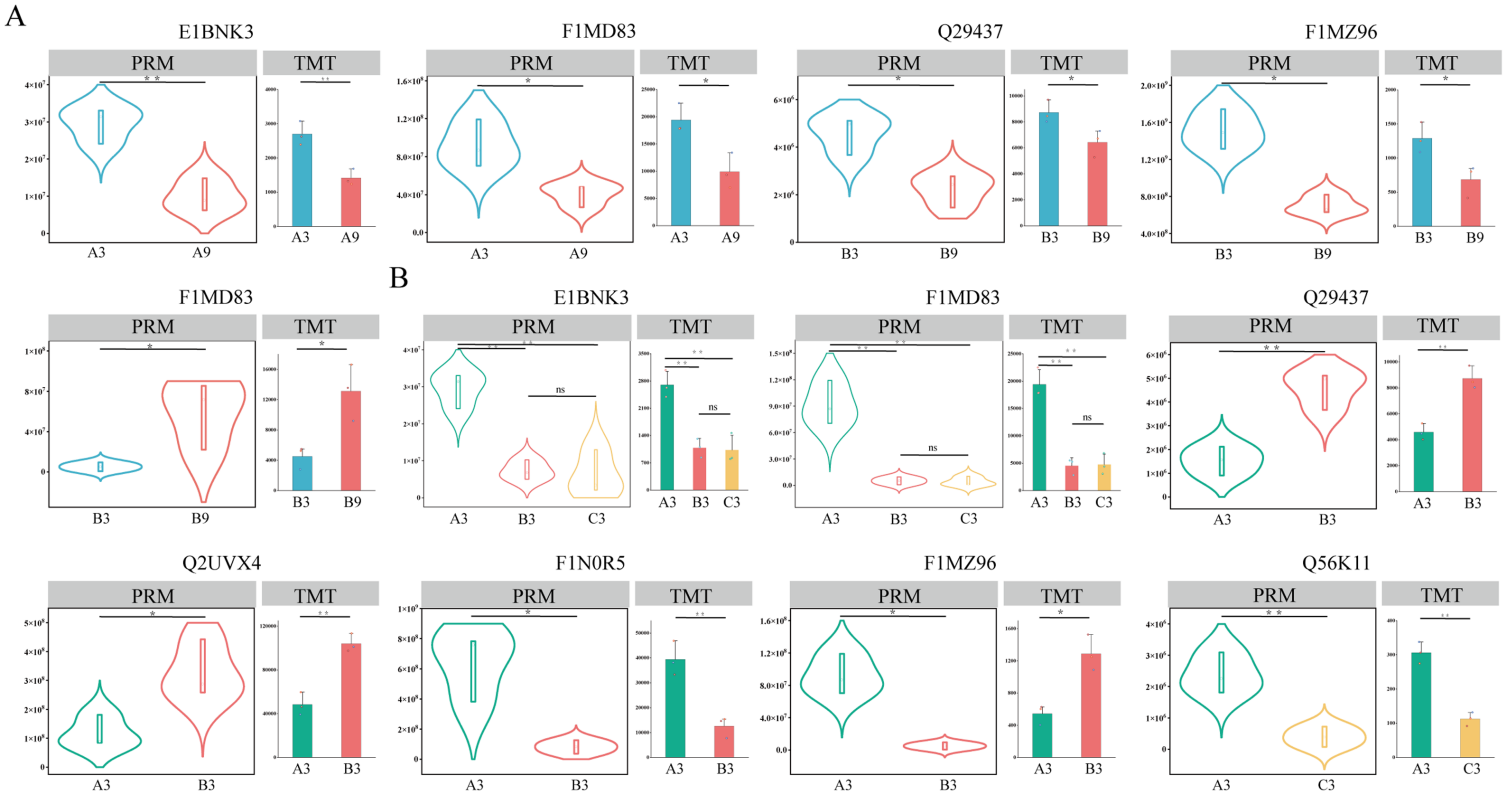
Validation of differential protein expression patterns identified by TMT analysis using parallel reaction monitoring (PRM). **(A)** TMT and PRM expression profiles of randomly selected differentially expressed proteins (DEPs) across age groups (3-month, blue, 9-month, red) within each generational cohort. **(B)** TMT and PRM expression profiles of randomly selected DEPs across generational groups (Generation A: green; Generation B: red; Generation C: yellow) within each age cohort.

To validate the differentially expressed proteins identified by LC–MS/MS analysis employing Tandem Mass Tag (TMT) technology, Parallel Reaction Monitoring (PRM) was implemented in this study for targeted verification. A total of twelve differentially expressed proteins were randomly selected from different comparison groups: five proteins from differing age cohorts (Figure 6A: E1BNK3, F1MD83 (A group), Q29437, F1MZ96, and F1MD83 (B group)) and seven proteins from different generational groups (Figure 6B: E1BNK3, F1MD83, Q29437, Q2UVX4, F1NOR5, F1MZ96, and Q56K11). The PRM results exhibited a high degree of concordance with the differential expression trends observed in the LC–MS/MS analysis, with statistical confirmation achieved for all twelve targeted proteins (12/12), thus supporting the reliability of the LC–MS/MS findings. In summary, these results demonstrate that the application of the TMT methodology in proteomic research provides robust accuracy and stability.

### 3.6 Protein–protein interaction network analysis

**Figure 7:**
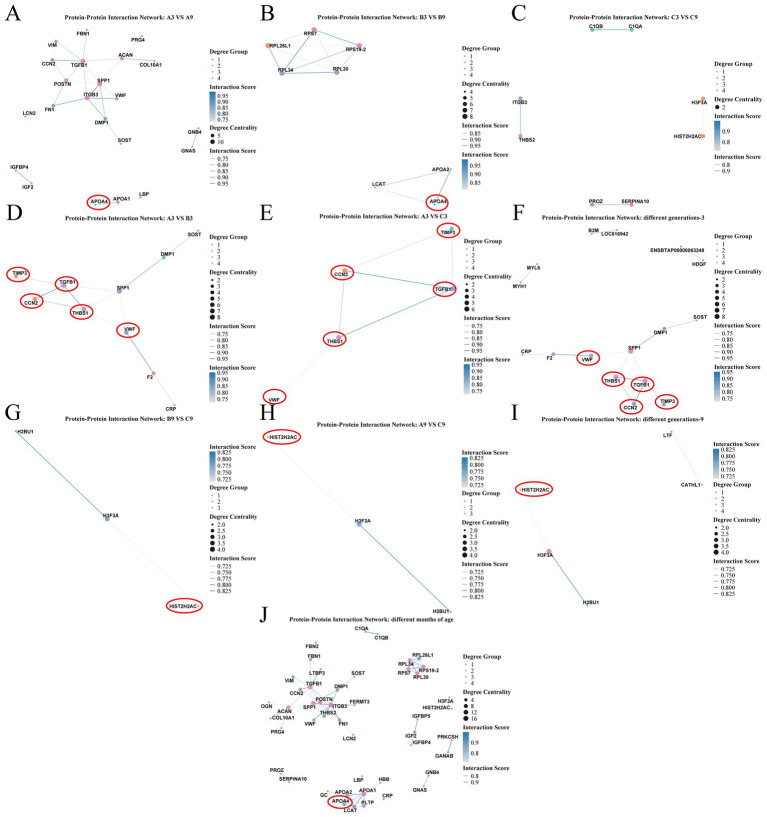
Protein–protein interaction (PPI) network analysis of differentially expressed proteins (DEPs) across age groups and generations in cattle. **(A–C)** PPI networks for age-group comparisons: A3 vs. A9, B3 vs. B9, and C3 vs. C9. **(D,E)** PPI networks for generational comparisons at 3 months: A3 vs. B3 and A3 vs. C3. **(G,H)** PPI networks for generational comparisons at 9 months: A9 vs. C9 and B9 vs. C9. **(F,I,J)** Comprehensive PPI networks integrating all DEPs: **(F)** 3-month generational groups, **(I)** 9-month generational groups, and **(J)** cross-age groups. Network parameters: Edge thickness and color denote interaction confidence score; Node color represents degree-based grouping; Node size corresponds to degree centrality. Proteins highlighted with red circles indicate shared DEPs across multiple comparison groups.

Using protein–protein interaction (PPI) network analysis, the functional interplay of differentially expressed proteins (DEPs) identified across different age cohorts and generational groups was investigated. A Comparative analysis of age cohorts (Figures 7A–C,J) revealed Apolipoprotein A-IV (APOA4) as a shared DEP. In both the A3 vs. A9 and B3 vs. B9 comparisons, APOA4 formed distinct interaction networks involving APOA1, LBP, APOA2, and LCAT proteins primarily related to lipid metabolism and cholesterol transport. However, significant interactions involving APOA4 were absent when comparing C3 vs. C9. Furthermore, within the comprehensive PPI network encompassing all DEPs, APOA4 also demonstrated interactions with PLTP, GC (Vitamin D-binding protein), CRP (C-reactive protein), and HBB (Hemoglobin subunit beta). This suggests its broader involvement in functions such as lipoprotein particle remodeling and the transport of triglyceride-rich lipoproteins.

In contrast, cross-generational comparisons revealed significant interactions only for a subset of DEPs, with no prominent networks observed in the B3 vs. C3 and A9 vs. B9 groups. Analysis of DEPs across generations at the 3-month stage (Figures 7D–F) identified Von Willebrand Factor (VWF), Thrombospondin-1 (THBS1), Cellular Communication Network Factor 2 (CCN2), Tissue Inhibitor of Metalloproteinase 3 (TIMP3), and Transforming Growth Factor Beta 1 (TGFB1) as shared DEPs in both the A3 vs. B3 and A3 vs. C3 comparisons. These proteins exhibited close interactions within the PPI network and are primarily associated with critical physiological processes such as immune regulation and growth/development. This suggests their potential synergistic role in early development and immune response across different cattle generations and provides crucial insights for further mechanistic investigations. At the 9-month stage (Figures 7G–I), Histone H2A Type 2-C (HIST2H2AC) was the key overlapping DEP across generations. It interacted with H3.3 Histone A (H3F3A) and Histone H2B Type U1 (H2BU1), forming a small network enriched in antibacterial responses and humoral immunity.

In summary, these PPI results describe distinct interaction patterns associated with age and generation. They reveal specific differences in inter-generational immune regulation and underscore the significant regulatory influence of genetic background on immune responses.

### 3.7 Screening of key differentially expressed proteins

**Figure 8:**
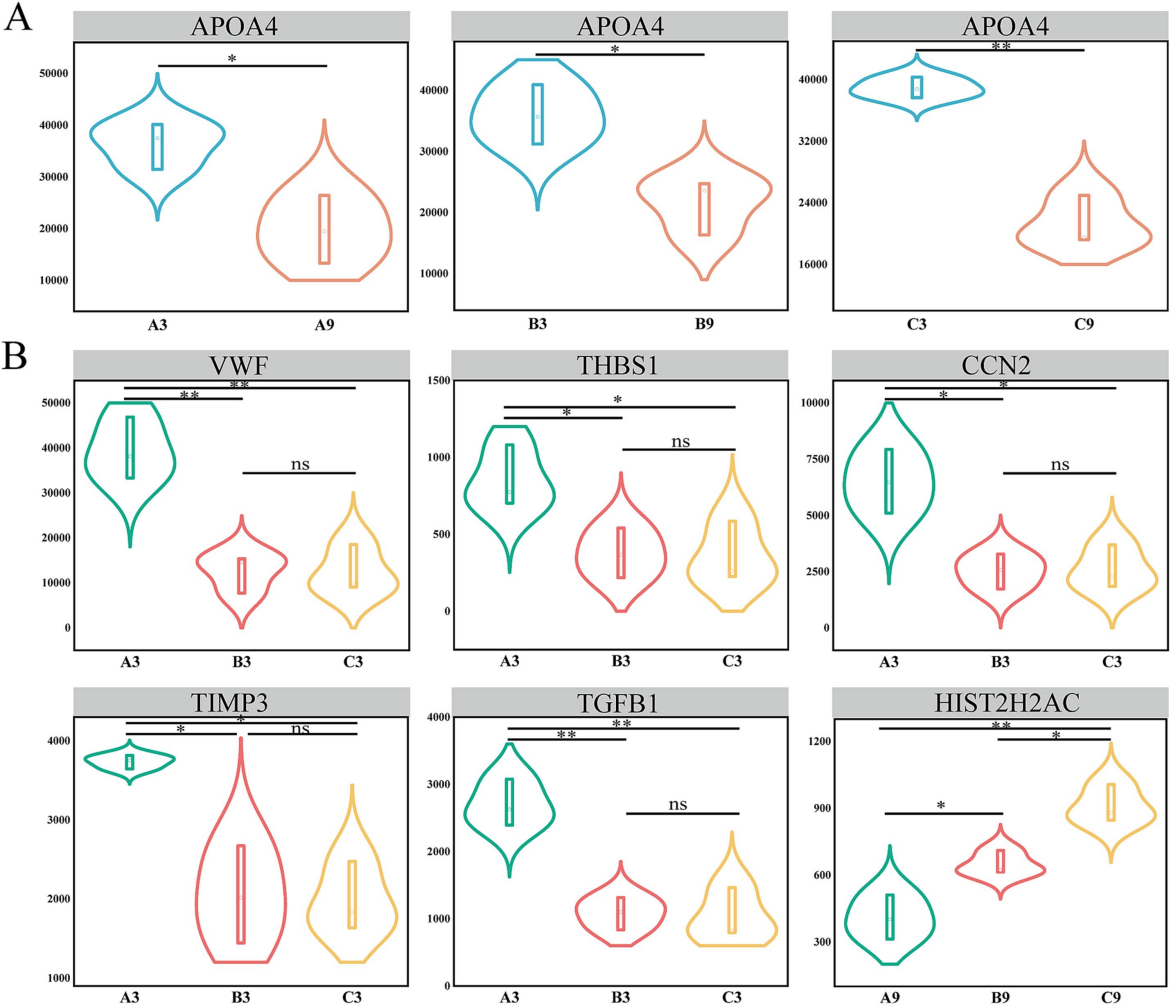
Expression dynamics of key differentially expressed proteins (DEPs). **(A)** Expression trends of APOA4 in age-group comparisons. **(B)** Expression trends of VWF, THBS1, CCN2, TIMP3, TGFB1, and HIST2H2AC in generational comparisons.

A Comprehensive analysis of differentially expressed proteins (DEPs) across age cohorts and generational groups identified seven key proteins such as, APOA4, VWF, THBS1, CCN2, TIMP3, TGFB1, and HIST2H2AC. These showed significant alterations in multiple comparisons and close interactions within the PPI network (Figure 8). Subsequent characterization revealed distinct expression dynamics and functional associations: APOA4 displayed consistent down regulation in all generational groups (A/B/C) at 9 months, with functional enrichment occurring predominantly in lipid metabolism pathways. VWF, THBS1, and TIMP3 showed significant down regulation in the 3-month intergenerational comparisons (B3/C3 vs. A3)and were collectively involved in platelet activation (GO:0030168) and cell adhesion (GO:0031589) while enriching for immune/inflammatory response pathways. In particular, CCN2 and TGFB1, which were similarly downregulated at 3 months, demonstrated functional convergence through heparin binding (GO:0008201) in chondrocyte differentiation (GO:0002062), MAPK signaling regulation (GO:0043406), and the TGF-*β* signaling pathway (ko04350), suggesting synergistic roles in tissue homeostasis and extracellular matrix remodeling. Crucially, HIST2H2AC, a key epigenetic regulator, showed dose-dependent upregulation across 9-month generational groups (C9 > B9 > A9) with functional enrichment in nucleosome assembly (GO:0000786), chromatin structural constitution (GO:0030527), and DNA binding (GO:0003677). KEGG analysis further implicated this histone in alcoholism (ko05034) and systemic lupus erythematosus (ko05322), possibly reflecting cumulative epigenetic modifications during genetic transmission (Supplementary Table S3).

## 4 Discussion

Using Integrated analysis of proteomics and bioinformatics. This study systematically identified key differentially expressed proteins and mapped their functional networks across different developmental stages and cattle generations in Xinjiang Brown cattle. Seven core regulatory proteins like APOA4, HIST2H2AC, TGFB1, VWF, THBS1, CCN2, and TIMP3, were found to form synergistic networks involved in lipid metabolism regulation, epigenetic adaptation, immune homeostasis, and skeletal development. These networks collectively shaping the genetic basis of growth performance and environmental adaptability in this breed. Our discussion integrates the key findings along three conceptual dimensions: metabolic reprogramming, epigenetic regulation, and immune-developmental trade-offs.

### 4.1 APOA4-driven lipid metabolic reprogramming and adaptability across growth stages

As a key member of the apolipoprotein family, apolipoprotein A4 (APOA4) is primarily synthesized in the intestine and plays a pivotal role in dietary lipid absorption, reverse cholesterol transport, and high-density lipoprotein (HDL) formation. It contributes to lipid transport and metabolic regulation (21, 22). Previous research have linked APOA4 expression with meat quality traits. For instance, in Limousin crossbred bulls, APOA4 was identified as a potential biomarker associated with beef sensory attributes such as tenderness, juiciness, and chewiness. Interestingly, upregulation of APOA4 were potentially linked to inferior meat quality, including reduced tenderness and juiciness (23). Furthermore, nutrition is recognized as a key factor influencing the serum protein profile of calves at different ages (24).

In the present study we observed that APOA4 expression was highly conserved across all three generations of Xinjiang Brown cattle (beef type). Expression levels were significantly higher in the 3-month-old group compared to the 9-month-old group. Functionally, APOA4 was enriched in lipid metabolism and cholesterol transport pathways. In ruminants, the efficiency of dietary lipid digestion and utilization is a key metabolic determinant, influenced by factors such as fat source and supplementation level, which can significantly alter rumen fermentation and overall nutrient digestibility (25). This pronounced age-dependent expression pattern of APOA4, considered alongside its role in lipid transport and the established importance of dietary fat utilization, positions it as a compelling biomarker that may link early-postnatal metabolic programming with later production traits.

This is Supported by evidence in mouse models, where deletion of apolipoprotein A4 (ApoA4) renders mice susceptible to high-fat diet (HFD)-induced non-alcoholic fatty liver disease (NAFLD) and insulin resistance. In contrast, liver-specific overexpression of ApoA4 or protein supplementation of ApoA4 protected against NAFLD and hepatic insulin resistance (26). Similarly, APOA4 interacts with the LRP1 receptor to promote glucose uptake via the PI3K-AKT signaling pathway. Down regulation of APOA4 expression impairs glucose uptake in mice, leading to insulin resistance, hyperglycemia, and dyslipidemia (27).

Dietary composition also modulates APOA4 expression. Zhong et al. (28) reported that sodium butyrate supplementation increased APOA4 protein expression in the intestinal epithelium of calves, along with concurrent alterations in lipid metabolism genes such as, APOA1 and APOA4. In Holstein dairy cows, essential fatty acids and conjugated linoleic acid also led to increased APOA4 expression, contributing to improved lipid metabolism and immune function (29).

Collectively, these findings suggest that the upregulation of APOA4 observed in 3-month-old Xinjiang Brown cattle (beef type) reflects an adaptive mechanism during the rapid growth phase. This mechanism enhances lipid metabolism and cholesterol transport to meet high energy demands. Consequently, APOA4 may serve as a biomarker for lipid utilization efficiency in young ruminants and presents a novel target for strategies aimed at improving calf growth performance. While our current data establish a strong correlative link, future studies investigating the direct functional impact of APOA4 modulation in cattle are warranted to elucidate causal mechanisms.

### 4.2 HIST2H2AC-mediated epigenetic memory and transgenerational adaptation

UHIST2H2AC (Histone Cluster 2 H2A Family Member C), a key variant of the histone H2A family, that showed a significant upward trend in expression in bovine serum (C9 > B9 > A9) across generations. This pattern reflects the cumulative effect of epigenetic modifications across generations. Since H2A variants are known for their low turnover in chromatin structure (30), HIST2H2AC may serve as a stable epigenetic marker that preserves environmentally or genetically induced modification patterns during generational transmission. Mechanistically, transgenerational selective pressures likely drive the intergenerational upregulation of HIST2H2AC by enhancing epigenetic memory through the EGFR-MEK/PI3K pathway (31, 32). Notably, structural studies on chromatin-associated proteins, reveal that specific domains coordinate to recognize DNA and stabilize nucleoprotein complexes, which is essential for maintaining chromatin architecture and genome stability (33). This suggests that HIST2H2AC, as a core histone variant, may similarly contribute to chromatin compaction and epigenetic memory through its structural integration into nucleosomes, thereby facilitating the heritability of expression patterns. Furthermore, altered HIST2H2AC expression is linked to DNA replication-coupled histone gene regulation. Elevated HIST2H2AC levels may indirectly stabilize epigenetic modifications by suppressing mTORC1 signaling or activating cell cycle inhibitors, both of which are associated with cell proliferation and growth (34, 35). Interestingly, functional enrichment analysis suggests that HIST2H2AC is involved in pathways related to immune dysregulation such as systemic lupus erythematosus, and metabolic stress responses like alcoholism, suggesting a broader role in maintaining bovine health. However, while HIST2H2AC upregulation may represent an adaptive epigenetic response, it may also have long-term adverse health effects. Dhahri et al. (36) found that silencing HIST2H2AC inhibits EGF-induced Zeb-1 expression and downregulates E-cadherin levels in mammary gland cells. This suggests that HIST2H2AC overexpression may promote tumorigenesis via epithelial-mesenchymal transition (EMT). This indicates that variants facilitating early adaptation may increase long-term pathological risks in cattle.

Therefore, future breeding strategies could benefit from integrating molecular marker screening to select animals with optimal expression profiles, thereby aiming to maximize production performance while mitigating potential long-term health compromises.

### 4.3 TGFB1-CCN2-TIMP3 network: evolutionary trade-off between immunity and development

In this study, we observed a coordinated down regulation of three key proteins, TGFB1, CCN2, and TIMP3 in the second and third generations of 3-month-old cattle. This trend suggests that artificial selection may have altered how these animals allocate resources between immune and developmental processes.

TGFB1 (Transforming Growth Factor Beta 1), a multifunctional cytokine belonging to the TGF-*β* superfamily, regulates cell proliferation, differentiation, metabolism, and signaling transduction. It is essential for maintaining tissue homeostasis, orchestrating development, and modulating immune responses. Previous studies demonstrate that TGF-β1 sustains immune homeostasis by suppressing IL-2 receptor expression via Smad2/3-dependent pathways to inhibit T-cell proliferation (37), while inducing Foxp3 expression to enhance regulatory T-cell (Treg) function (38). Given the controlled breeding environment and the transgenerational nature of the observed downregulation, it is unlikely to be solely due to environmental fluctuations within each generation. Instead, this pattern strongly points towards heritable epigenetic reprogramming as the primary driver. This aligns with our finding of upregulated HIST2H2AC, a core histone variant, suggesting a selection-driven stabilization of repressive chromatin states that could propagate TGFB1 silencing across generations. In Tgfb1-knockout mice, systemic immune hyperactivation causes fatal multiorgan inflammation, confirming its non-redundant immunosuppressive role (6). In addition to its immune function, TGF-β1 suppresses tissue damage during acute inflammation by inhibiting proinflammatory cytokine release. For instance, exogenous TGF-β1 attenuates ventilator-induced lung injury by reducing neutrophil infiltration and IL-1*β* levels (39). Notably, TGF-β1 also mediates cellular differentiation processes: In equine endometrium, it induces myofibroblast differentiation, ECM overproduction, and fibroblast proliferation, contributing to pathological fibrosis (40). Concurrently, it prevents dystrophic muscle fibroblast apoptosis and cell cycle arrest through canonical NF-κB signaling (41).

Our results suggest that first-generation cattle may have benefited from elevated TGFB1 expression as an early adaptive strategy to reinforce immune homeostasis, accelerating skeletal development (via chondrogenesis and bone remodeling), and optimizing energy metabolism (through ATP synthesis and phosphoinositide signaling). It prioritizes survival-critical functions to cope with high pathogen exposure and nutritional fluctuations in natural environments. In contrast, the down regulation in TGFB1 expression in later generations may reflect a breeding prioritization of muscle growth and feed efficiency, beneficial for production traits but may also increase susceptibility to immune dysregulation or fibrosis.

CCN2 (Cellular Communication Network Factor 2), a core member of the CCN family, dynamically regulates chondro-osteogenic conversion and extracellular matrix (ECM) homeostasis through synergistic interactions with integrin/HSPG signaling (42, 43). It is indispensable for osteoblast differentiation and bone matrix mineralization; CCN2-deficient mice exhibit severe skeletal defects (44). The down regulation of CCN2 in B3/C3 generations observed here may thus impede skeletal development. Furthermore, as a downstream effector of TGFB1 (45) and a “molecular tuner” of TGF-*β*/BMP signaling, CCN2 enhances BMP-4 osteogenic activity while suppressing TGF-β1-driven fibrogenesis via its cysteine-rich (CR) domain (46). This dual regulation implies that reduced CCN2 expression may alleviate fibrotic phenotypes in muscle and other tissues by inhibiting TGF-β/Smad-mediated collagen deposition (47, 48).

In summary, CCN2 down regulation likely reflects an adaptive reallocation of energy resources by attenuating energy-intensive bone formation and fibrotic repair. Given CCN2’s critical role in bone mineralization (44), its sustained downregulation could predispose animals to compromised bone integrity or fragility over the long term, representing a potential trade-off for enhanced metabolic efficiency directed toward muscle growth. This concept of nutrient partitioning is supported by studies in ruminant nutrition. For instance, Truong et al. (49) demonstrated that reducing dietary neutral detergent fiber (NDF) from 55 to 43% significantly improved dry matter intake, nutrient digestibility, and daily weight gain in Charolais crossbred cattle. This highlights how dietary composition directly influences metabolic efficiency and growth outcomes. In our context, selective breeding for traits like feed efficiency may systemically favor metabolic programs that divert resources away from “costly” processes like high-fidelity bone remodeling towards prioritized muscle deposition.

TIMP3 (Tissue Inhibitor of Metalloproteinase 3) is unique among the members of the TIMP family in that it binds directly to ECM components and blocks TACE/ADAM17 activity, thereby preventing the release of soluble TNF-*α*-converting enzyme (50). Our generational data suggest a steady decline in TIMP3 levels, which could be caused by epigenetic silencing for example, promoter hypermethylation has been shown to suppress TIMP3 in cancer (51, 52). In human cancers (e.g., meningioma, ovarian carcinoma), environmental stressors or hereditary factors drive progressive TIMP3 methylation accumulation across generations or disease stages (53, 54). Demethylating agents (e.g., 5-Aza-2′-deoxycytidine) restore TIMP3 expression (55), suggesting that targeted breeding strategies in cattle may similarly propagate methylation-mediated TIMP3 suppression. In addition to epigenetic regulation, TIMP3 inhibits soluble TNF-α release by blocking TACE activity, thereby suppressing inflammation (56). Its deficiency exacerbates adipose inflammation and insulin resistance via the TACE-TNF-α axis in metabolic disorder models (57, 58).

Thus, transgenerational TIMP3 down regulation likely arises from environment-epigenetic-metabolism interactions. Future breeding programs should incorporate an immune-metabolic balance index to select individuals with optimal growth performance and stress resilience using multi-omics screening approaches.

### 4.4 VWF-THBS1 axis: breeding trade-offs in hemostasis-inflammation regulation

Compared to the first-generation Xinjiang Brown cattle (beef type) at 3 months, significant down regulation of von Willebrand factor (VWF) and thrombospondin-1 (THBS1) in later generations reflects artificial selection’s impact on vascular homeostasis. VWF, a high-molecular-weight multimeric glycoprotein synthesized by endothelial cells and megakaryocytes, coordinates hemostasis through its A1 domain (binding platelet GPIbα for adhesion), A2 domain (containing ADAMTS13 cleavage site Tyr^1605^-Met^1606^ to limit prothrombotic activity), and A3 domain (anchoring to collagen III at injury sites) (59, 60). Beyond hemostasis, VWF mediates inflammation and vascular repair via Weibel-Palade body release of P-selectin and chemokines (61, 62). In this study, VWF down regulation with concurrent enrichment in platelet activation (GO:0030168) and intracellular signaling regulation (GO:1902533) suggests an adaptive trade-off favoring production traits at the cost of diminished platelet adhesion, tissue repair, and inflammatory responsiveness. However, this interpretation would be strengthened by integration with physiological data, such as clotting times or platelet function assays, to directly link the observed proteomic shift to tangible hemostatic phenotypes in these cattle.

THBS1 modulates immune tolerance through CD47 binding (inhibiting CD8^+^ T-cell function and macrophage phagocytosis) and TGF-*β*1 activation (driving Treg differentiation while suppressing Th1/Th17 cytokines) (63, 64). At the same time, THBS1 exhibits context-dependent proinflammatory effects, as demonstrated by its exacerbation of LPS-induced lung inflammation and oxidative stress in pneumonia models (65). The observed generational THBS1 suppression may thus attenuate TGF-β-dependent fibrosis and tumor immune evasion while reducing pathological inflammation. This immune-vascular adaptation is particularly relevant in the context of disease resistance, as evidenced by epidemiological studies linking immune-vascular dysregulation with increased susceptibility to infectious diseases such as lumpy skin disease (LSD), where seroprevalence and risk factors correlate with immune and inflammatory status in cattle populations (66). Furthermore, the modulation of inflammatory pathways through natural compounds, as reviewed by Iqbal et al. (67), highlights the potential for targeted immune resilience strategies in breeding programs aimed at enhancing disease resistance without compromising growth traits. It is important to note that while this study was conducted on a farm with standardized management protocols, subtle variations in environmental factors such as feed composition over the years could represent uncontrolled variables. However, the progressive, generation-dependent trends observed in key proteins suggest that the signature of artificial selection is a predominant driver of the proteomic shifts. Nevertheless, the precise mechanisms underlying this developmental homeostasis remain to be fully elucidated and warrant further investigation.

## 5 Conclusion

This serum proteomic analysis reveals that generational selection in Xinjiang Brown cattle drives adaptive changes in key proteins involved in lipid metabolism (APOA4), immune regulation (HIST2H2AC, TGFB1 network), and hemostasis-inflammation balance (VWF/THBS1). These findings highlight potential trade-offs between growth performance and immune-vascular resilience, supported by epidemiological relevance to disease susceptibility in cattle populations. While providing novel molecular insights for precision breeding, this study has limitations, including its correlative nature, the modest sample size per group, and the lack of *in vivo* validation. Future work should integrate physiological and disease-challenge models with larger cohorts to validate these proteomic markers and their functional implications for sustainable cattle improvement.

## Data Availability

The mass spectrometry proteomics data have been deposited to the ProteomeXchange Consortium (https://proteomecentral.proteomexchange.org) via the iProX partner repository (68, 69) with the dataset identifier PXD069377.

## Supplementary material

SUPPLEMENTARY FIGURE 1 Protein quality results. **(A)** Standard curve for protein concentration. **(B)** SDS-PAGE (polyacrylamide gel) electrophoresis test.
