# Supplementary figures and images for: TMT-based proteomic profiling of serum reveals the impact of developmental stage and generation in beef cattle
Source: Front Vet Sci. 2026 Mar 9;13:1723813. doi: 10.3389/fvets.2026.1723813 (PMC13007506; doi:10.3389/fvets.2026.1723813)

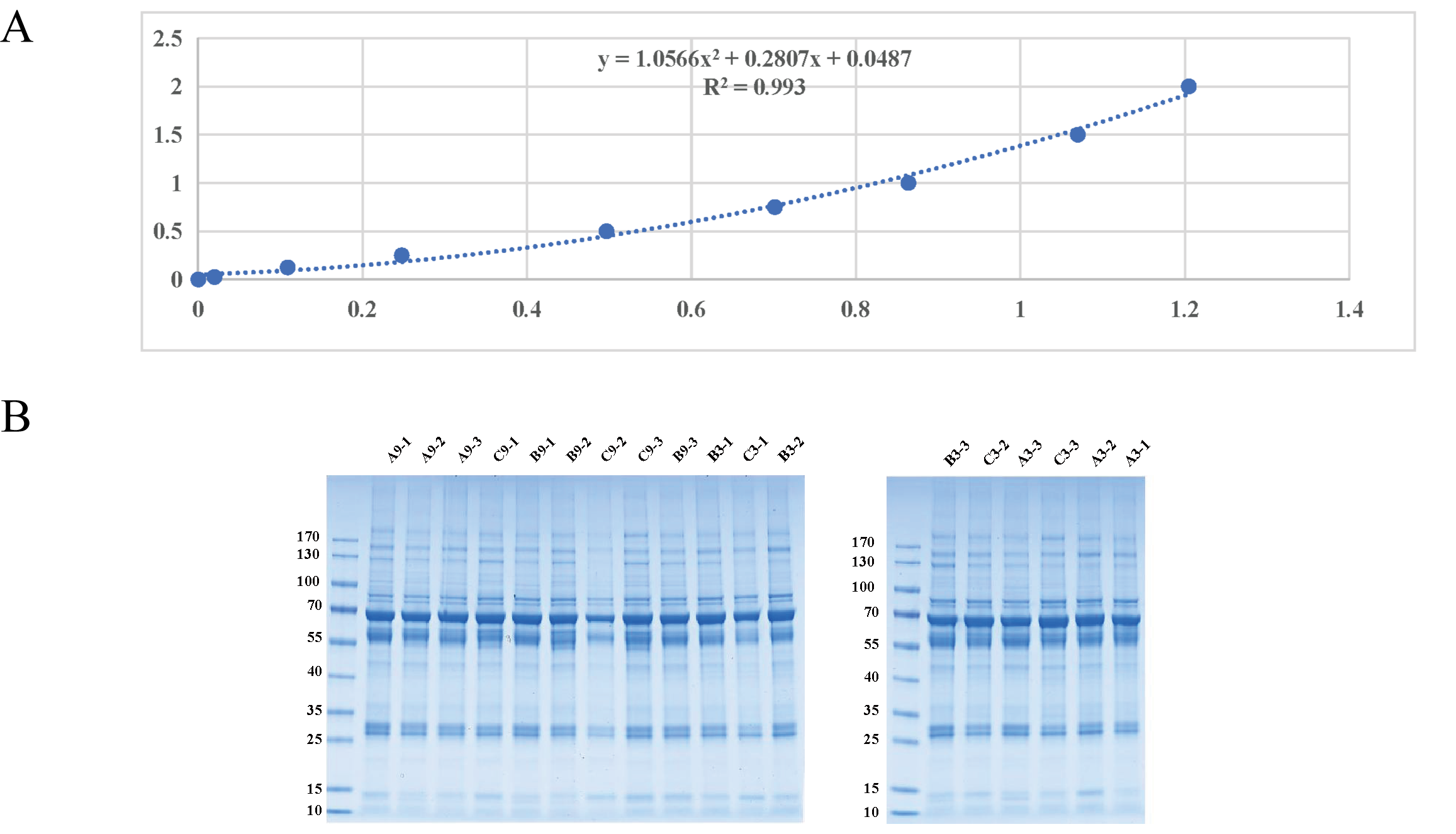

Supplement: SUPPLEMENTARY FIGURE 1 — Protein quality results. (A) Standard curve for protein concentration. (B) SDS-PAGE (polyacrylamide gel) electrophoresis test. [file Image_1.TIF]
